# A Case of Neuroma of the External Popliteal Nerve

**Published:** 1894-03-24

**Authors:** A. G. Miller

**Affiliations:** Senior Surgeon to the Edinburgh Royal Infirmary


### A CASE OF NEUROMA OF THE EXTERNAL POPLITEAL NERVE.

By A. G. Miller, M.D., F.R.C.S.E., Senior Surgeon to the Edinburgh Royal Infirmary.

I would ask attention of the following points in this case: (1) The coincidence of pain with an injury. Was the growth of the neuroma the direct result of the strain? (2) The sudden appearance of the paralysis a few days (six or seven) after the operation. (3) This paralysis due to inflammation (neuritis). The nerve though adherent to the cicatrix was not compressed, in fact, it was larger than normal. (4) Complete recovery under rest, counter-irritation, and the constant current.

Elizabeth H——, aged 29, a domestic servant, and unmarried, was admitted December 11th, 1891, to the Edinburgh Royal Infirmary for a swelling a little below the left knee, causing intense pain.

The history was as follows: About nine months previous to admission the patient noticed that her left leg became tired sooner than her right one, and that after any excessive exertion it was swollen, stiff, and painful. She paid no attention to this, attributing it to the result of her extra work. About six months ago the leg was attacked by shooting pains passing from behind the knee down the front of the leg to the dorsum of the foot. She had the leg bathed with hot water and rubbed with embrocation, and noticed that the most painful part when rubbed was behind the knee. Dr. Brackenridge, whom she consulted, advised her to paint the part with iodine. This application soothed the pain for a time, but the slightest knock made the part as painful as ever. She now noticed a small swelling at the seat of the pain, and on her second visit to Dr. Brackenridge on November 11th, he advised her to consult Mr. Miller. No previous illnesses, but an accident nine months ago, viz., a fall which injured her back and twisted her left leg. Her mode of life has always been healthy, and she has always been temperate in both food and drink. Her father and mother are both alive and well. She has brothers and sisters, all of whom are strong, and the health of her other relations is satisfactory. On examining the affected leg a somewhat oval swelling is noticed over the head of the left fibula, and extending slightly backwards into the popliteal space; it is tender to the touch and excessively painful on compression. The general sensation of both legs is normal. The pain she complains of has been getting gradually worse. It did not hinder movement at first, but of late the movement of the knee has been restricted by the pain. The size of the swelling has not noticeably increased. The pain is always worse at night, and after any exertion, and the leg also swells at night. A diagnosis of neuroma of the external popliteal nerve was made.

On December 15th, 1891, the patient was chloroformed, the limb rendered bloodless, and an incision about 2½ inches long was made over the tumour, dividing the superficial structures only. The tumour was then carefully dissected down upon. It was found encapsuled, of a bluish colour, and adherent at the upper part to the external popliteal nerve, some of the nerve fibres being spread out over it at this point. At the lower part the tumour was attached also to the nerve, but the fibres were not spread over it as above. Some of the fibres of the nerve at the upper part were unavoidably removed along with the tumour. During dissection the capsule was punctured and a gelatinous material exuded. On examination the tumour was found to be a soft myxoma. No vessels were ligatured. A deep silk suture was put in, and a continuous silk suture used to bring the superficial edges of the wound together. No drain was employed. The wound healed by first intention.

About a week after the operation the foot began to drop and was much swollen. A splint was applied to keep it in position, and massage and electricity were used daily to prevent degeneration of the muscles.

On February 9th, under chloroform, a second operation was performed. An oblique incision, running downwards and forwards over the head of the fibula, was made, and the cicatrix of the former operation included in this incision. The tissues were found to be dense and cicatrised. What at first appeared to be a piece of fat was found to be a branch of the external popliteal nerve, evidently divided at the former operation. The trunk of the nerve was found surrounded by, and firmly adherent to, cicatricial tissue, and on cleaning it the nerve was seen to be softened and inflamed at one part. The peroneus tongue was divided to a slight extent at its outer border in order to trace the lower end of the divided branch already referred to. The two ends were sutured by a single fine catgut suture, the upper end having been refreshed by the knife. The wound was then dusted with boric powder, stitched with horsehair (a protective tissue drain being inserted), and dressed with wood wool wadding. The part was blistered several times during March.

On April 5th the constant current was applied, one pole of the battery being placed above and behind the knee, and the other at the various motor points on the leg; and this treatment was followed daily. When the current was first employed there was almost complete absence of power in the flexors of the foot, and in the peronei muscles. The foot and toes pointed, and there was slight inversion. After the constant current had been employed two days slight power of dorsiflexion was regained in the foot, and the great toe could be slightly extended, this having been previously impossible. In a week there was a marked improvement, and patient was discharged on April 15th, with directions to use and massage her foot daily. She returned to show herself about three weeks after this date, and had regained still more the use of the affected muscles, being now able to turn up her toes pretty well, and to evert the foot to some extent.

June 25th: Patient reported herself. The movements of the foot are all perfectly restored; she can walk quite well. Slight weakness of the muscles of leg and foot is all that can be noted as abnormal.

November 15th: Patient reported herself. She is now perfectly well, and able to perform her household duties.

---

